# Performance of different Dixon-based methods for MR liver iron assessment in comparison to a biopsy-validated R2* relaxometry method

**DOI:** 10.1007/s00330-020-07291-w

**Published:** 2020-09-23

**Authors:** Benjamin Henninger, Michaela Plaikner, Heinz Zoller, André Viveiros, Stephan Kannengiesser, Werner Jaschke, Christian Kremser

**Affiliations:** 1 Department of Radiology, Medical University of Innsbruck, Anichstraße 35, 6020 Innsbruck, Austria; 2 Department of Internal Medicine, Medical University of Innsbruck, Innsbruck, Austria; 3 MR Application Development, Siemens Healthcare GmbH, Erlangen, Germany

**Keywords:** Dixon, Relaxometry, Iron, Liver, Magnetic resonance imaging

## Abstract

**Objectives:**

To prospectively evaluate a 3D-multiecho-Dixon sequence with inline calculation of proton density fat fraction (PDFF) and R2* (qDixon), and an improved version of it (qDixon-WIP), for the MR-quantification of hepatic iron in a clinical setting.

**Methods:**

Patients with increased serum ferritin underwent 1.5-T MRI of the liver for the evaluation of hepatic iron overload. The imaging protocol for R2* quantification included as follows: (1) a validated, 2D multigradient-echo sequence (initial TE 0.99 ms, R2*-ME-GRE), (2) a 3D-multiecho-Dixon sequence with inline calculation of PDFF and R2* (initial TE 2.38 ms, R2*-qDixon), and optionally (3) a prototype (works-in-progress, WIP) version of the latter (initial TE 1.04 ms, R2*-qDixon-WIP) with improved water/fat separation and noise-corrected parameter fitting. For all sequences, three manually co-registered regions of interest (ROIs) were placed in the liver. R2* values were compared and linear regression analysis and Bland-Altman plots calculated.

**Results:**

Forty-six out of 415 patients showed fat-water (F/W) swap with qDixon and were excluded. A total of 369 patients (mean age 52 years) were included; in 203/369, the optional qDixon-WIP was acquired, which showed no F/W swaps. A strong correlation was found between R2*-ME-GRE and R2*-qDixon (*r*^2^ = 0.92, *p* < 0.001) with Bland-Altman revealing a mean difference of − 3.82 1/s (SD = 21.26 1/s). Correlation between R2*-GRE-ME and R2*-qDixon-WIP was *r*^2^ = 0.95 (*p* < 0.001) with Bland-Altman showing a mean difference of − 0.125 1/s (SD = 30.667 1/s).

**Conclusions:**

The 3D-multiecho-Dixon sequence is a reliable tool to quantify hepatic iron. Results are comparable with established relaxometry methods. Improvements to the original implementation eliminate occasional F/W swaps and limitations regarding maximum R2* values.

**Key Points:**

*• The 3D-multiecho-Dixon sequence for 1.5 T is a reliable tool to quantify hepatic iron.*

*• Results of the 3D-multiecho-Dixon sequence are comparable with established relaxometry methods.*

*• An improved version of the 3D-multiecho-Dixon sequence eliminates minor drawbacks.*

## Introduction

In recent years, magnetic resonance imaging (MRI) has been established for the evaluation of hepatic iron overload [1, 2]. The benefits of MRI are at hand: non-invasive, nowadays widely available, no relevant risk factors, additional information on iron overload of the spleen and pancreas, reduction of sampling errors to a minimum [3–5].

Nevertheless, there are also some limitations that have been addressed in the last years. One problem is the wide range of different techniques, as e.g. R2 or R2* relaxometry or the signal-intensity-ratio method [6–9]. Further, most of these approaches do not have regulatory approval for iron quantification, which limits their use in larger multicenter studies or clinical trials. Small institutions or private practices without the availability of experts in the field mostly do not provide iron quantification due to the seemingly complex sequences and post-processing procedures. In addition, the variety of different measurement sequences and software solutions complicates the comparability of the various methods. Consensus is still missing, which makes it even more difficult for each institution to find the best approach.

Most vendors of MR scanners have recently developed Dixon-based solutions with integrated post-processing where PDFF and R2* are simultaneously calculated [10], and may be used for quantifying iron [11]. The corresponding products for different vendors are thereby known under the following brand names: “IDEAL-IQ” from General Electric, “StarQuant” (or mDixon-Quant) from Philips, and “LiverLab” (or qDixon) from Siemens Healthcare. These sequence techniques are promising to fulfill the requirements for an accurate evaluation of iron, however, with rather high purchase prices. In the literature, there is only limited data on the clinical usefulness and accuracy of these approaches [11–13]. The qDixon sequence used in our institution is based on a 3D multigradient-echo acquisition and uses controlled aliasing undersampling [14], which allows acquisition in a single breath-hold. Further, advanced inline processing via a multistep adaptive fitting approach facilitates evaluation without further post-processing [11]. Any image-viewing software, that allows region of interest (ROI)–based signal intensity measurements, can be used for measuring R2* and proton density fat fraction (PDFF) values.

As studies evaluating clinical applications of commercial Dixon-based sequences for hepatic iron quantification are rare, it was the purpose of our study to evaluate qDixon and an improved (works-in-progress) version of this sequence (qDixon-WIP) for the assessment of hepatic iron overload in daily clinical routine to enhance confidence in these methods. For this purpose, we compared results from qDixon/qDixon-WIP with an established, biopsy-calibrated 2D multiecho R2* relaxometry method [9].

## Materials and methods

This prospective study was approved by our Institutional Review Board (Medical University of Innsbruck). Written informed consent was obtained from each patient.

### Patients

All patients were referred to our department (Department of Radiology, Medical University of Innsbruck) for the evaluation of hepatic iron overload between December 2015 and September 2019. The inclusion criteria were as follows: (1) increased serum ferritin (> 300 μg/L in male patients and > 200 μg/L in female patients, (2) age > 18 years, (3) acquisition of our MRI protocol for the evaluation of diffuse liver disease as listed below, where qDixon-WIP was available only from November 2017 and therefore an optional sequence. General contraindications to MRI were used as exclusion criteria. Further, patients that showed a complete fat/water swap (F/W swap) at the qDixon sequence were not included in our study.

### MR examination and image analysis

All patients were examined with a 1.5-T whole-body MR scanner (MAGNETOM Avanto^fit^, Siemens Healthcare). Patients were scanned in supine position using an 18-element body matrix coil and 12–16 elements of the integrated 32-channel spine matrix coil. The technicians carefully instructed the patients to suspend respiration at end expiration and to be consistent in their breath-holds. Our protocol for diffuse liver disease is provided in Table 1. We aimed at evaluating three sequences, which are relevant for the quantification of hepatic iron: qDixon, qDixon-WIP, and our reference sequence R2*-ME-GRE. Each sequence was acquired in breath-hold and in transversal orientation. For the comparison between the sequences, R2*-ME-GRE was considered reference because it was already evaluated in a clinical setting and correlated to biopsy data in earlier studies [9]. The qDixon sequence automatically calculates PDFF and R2* maps during image reconstruction without the need of further post-processing. Though the sequence is focused on the quantification of liver fat fraction, the sequence parameters suggested by the vendor (in particular the long initial echo time) were, not changed for this study, which would also be the case in small institutions or private practices without special technical expertise in the field.

**Table 1:** MR imaging protocol

	R2*-ME-GRE	qDixon	qDixon-WIP (optional)
Initial TE (ms)	0.99	2.38	1.04
Number of echoes (*n*)	12	6	6
Delta TE (ms)	1.41	2.38	1.17
Max. TE (ms)	16.5	14.28	6.89
TR (ms)	200	15.6	9
Flip angle (°)	20	4	4
Receive bandwidth (Hz/Px)	1955	1080	1080
Total acceleration factor	-	4	3
Matrix (mm)	128 × 128	160 × 136	160 × 120
Field of view	360–380	360–380	360–380
Slice thickness (mm)	10	3.5	3.5
Number of slices	2^1^ (two breath-holds)	64 (one breath-hold)	80 (one breath-hold)
Fat saturation	CHESS^2^	Dixon	Dixon
Data acquisition/type of sequence (2D/3D)	2D	3D	3D
Acquisition time (s)	16.8	18.51	16.37

^1^Single slice through the liver in two different slice positions

^2^Chemical-shift selective fat saturation (as provided by the manufacturer)

qDixon-WIP is a prototype version with the same MR sequence part as for the qDixon product sequence, however, with several improvements integrated into the inline image reconstruction: global fat/water (F/W) swaps during the initial Dixon water/fat separation stage of the multistep fitting approach [10] are detected using an AI-based classificator [15] and reversed if necessary. To mitigate noise bias in the subsequent magnitude fitting stage, a noise map is calculated. It is based on the system’s built-in adjustment functionality, which measures noise for the given receive coil setup, in combination with knowledge about the noise propagation through the individual image reconstruction steps as described in [16]. First-moment noise-corrected parameter fitting is then performed analogous to the approach described in [17], but with the noise level being a value known via the noise map, rather than a free parameter of the signal model. Also, the fat signal dephasing term is retained in the signal model, which then reads

$$ \left|{s}_{\mathrm{n}}\right|={E}_{\sigma}\left\{\left|\left(w+{c}_{\mathrm{n}}\cdotp f\right)\ \exp \left(-{R}_2^{\ast}\cdotp {TE}_{\mathrm{n}}\right)\right|\right\}\cdotp $$

|*s*_n_| is the magnitude signal measured at echo time *TE*_n_, *w* and *f* are the (unknown) water and fat signal components, respectively, and *c*_n_ is the complex-valued fat signal dephasing factor at echo time *TE*_n_. *E*_*σ*_{…} denotes the expectation value of the term in brackets given the (known) noise level *σ*. Finally, an additional inline calculation of liver iron concentration (LIC) maps was implemented, which allows ROI measurements in iron units. In addition to the modified inline image reconstruction, the initial TE and ΔTE were reduced for qDixon-WIP to 1.04 ms and 1.17 ms, respectively, without changes of receive bandwidth. The reduced TE values subsequently lead to a decrease of TR which could be exploited to reduce the total acceleration factor while still obtaining a slightly shorter acquisition time (Table 1).

R2* maps for the R2*-ME-GRE sequence were calculated using a custom-written plugin for ImageJ (Wayne Rasband, National Institutes of Health) by fitting on a pixel-wise basis with a truncation model [18]. For image analysis of qDixon and qDixon-WIP, our local picture archiving and communication system (PACS) was used (IMPAX; Agfa-Gevaert). Image analysis was performed independently by a radiologist (P.M.) with 9 years of experience in liver MRI (ROI placement) and by a physicist (C.K.) with 14 years of experience in liver MRI post-processing (calculation of the R2* maps). First, the liver was reviewed concerning possible focal liver lesions or artifacts. Then, three manually co-registered regions of interest (ROIs) were placed within the liver for all sequences, two in the right lobe and one in the left lobe. Major vessels were avoided. The diameter was 10–13 mm with an area of 0.72–1.15 cm^2^. The mean R2* value (1/s) was calculated using the available three ROI measurements.

Further, we calculated the LIC for qDixon using a cross-calibration with the reference R2*-ME-GRE sequence and additionally correlated the obtained results using different available calibration equations from studies by Wood et al, Henninger et al, Hankins et al and Garbowski et al. [6, 9, 19, 20]. Agreement between all LIC results was calculated based on direct LIC values and based on two different evaluation criteria: (1) a simple iron yes/no classification defined by a LIC of > 36 μmol/g (2 mg/g) and (2) based on the classification system proposed by the EASL [21].

### Statistical analysis

Statistical calculations were performed using the R Project for Statistical Computing [22]. To analyze the correlation and agreement between the different methods, the mean value of the three measured ROIs within the liver was used for each patient. Linear regression analysis was performed by fitting a linear model to the data, and Bland-Altman plots were calculated to visualize the agreement between the respective methods. In addition to Bland-Altman plots, Lin’s concordance correlation coefficient [23] was calculated to assess the degree of agreement between methods using the epiR package for R [24]. Concordance correlation coefficients were rated as follows: < 0.9: poor agreement; 0.9–0.95: moderate agreement; 0.95–0.99: substantial agreement; > 0.99: almost perfect agreement. To determine the agreement of iron classification based on different published calibration data, contingency tables between pairs of these calibrations were generated and Cohen’s kappa coefficient with equal weights was calculated using the rel package for R [25].

## Results

Forty-six out of 415 patients showed a F/W swap with qDixon and were therefore excluded. A total of 369 patients (283 males, 86 females, mean age 52 years, range 18–82 years) were prospectively included in our study. In 203/369 patients, the optional qDixon-WIP sequence was also acquired. No F/W swap was encountered with the qDixon-WIP in any of the 203 patients.

A drawback of the qDixon sequence is that it seems to be limited to a maximum R2* value of around 400 1/s. For the qDixon-WIP sequence, no such limitation was observed.

R2* values with qDixon ranged from 21.6 to 441.3 1/s (mean 81.7 1/s), with qDixon-WIP from 25.8 to 668 1/s (mean 76.9 1/s) and with R2*-ME-GRE from 24.6 to 571.8 1/s (mean 85.5 1/s).

**Fig. 1:**
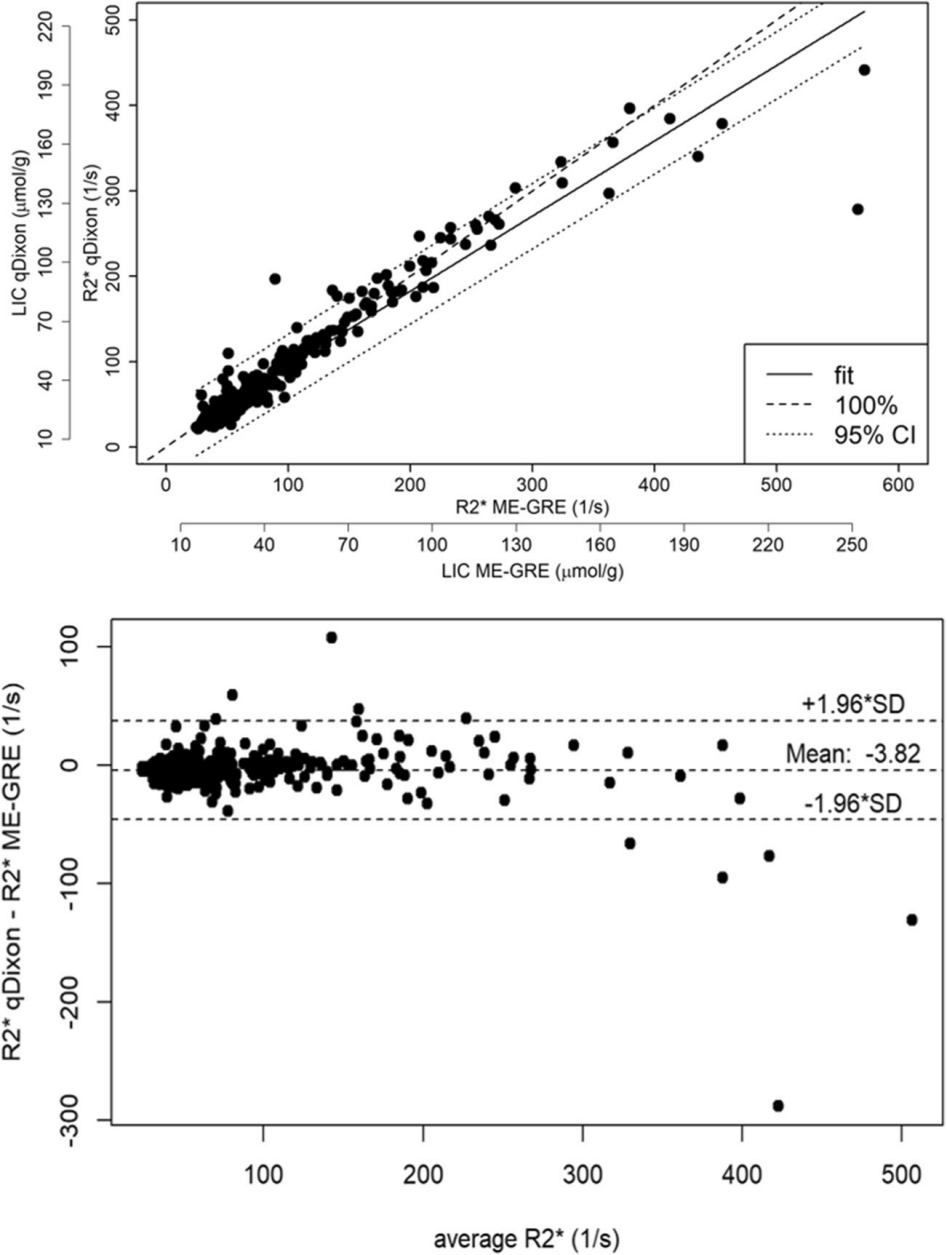
Correlation between liver R2* of R2*-ME-GRE and qDixon (top) and corresponding Bland-Altman plot (bottom) (mean difference = − 3.82; SD = 21.26; concordance correlation coefficient: 0.955). The qDixon sequence is limited to maximum R2* values of about 400 1/s. The additional axis in the upper part of the figure enables the quantification of LIC based on the respective calibration equations given in the “Results” section

Correlation analysis between R2* values of qDixon and R2*-ME-GRE for all patients showed an *R*^2^ of 0.92 (*p* < 0.05). Bland-Altman analysis revealed no systematic effect in the difference of R2* values between both sequences (mean = − 3.82; SD = 21.26) (Fig. 1), and a concordance correlation coefficient of 0.955 (range: 0.946–0.963) revealed substantial agreement. Taking into account that R2* of qDixon seems to be limited to R_2_^*^ values of around 400 1/s, correlation analysis only for patients with R2* ≤ 400 1/s showed an *R*^2^ of 0.956 (*p* < 0.05) with linear regression giving a relationship of R_2_^*qDixon^ = 1.00564 * R_2_^*ME-GRE^ − 2.7. From the known LIC calibration equation for R_2_^*^-ME-GRE [9], we obtain the following calibration equation for qDixon: Fe (μmol/g) = 0.434 * R_2_^*^ + 6.135. In a similar manner, the calibration equation for qDixon-WIP was found to be Fe (μmol/g) = 0.429 * R_2_^*^ + 5.682.

**Fig. 2:**
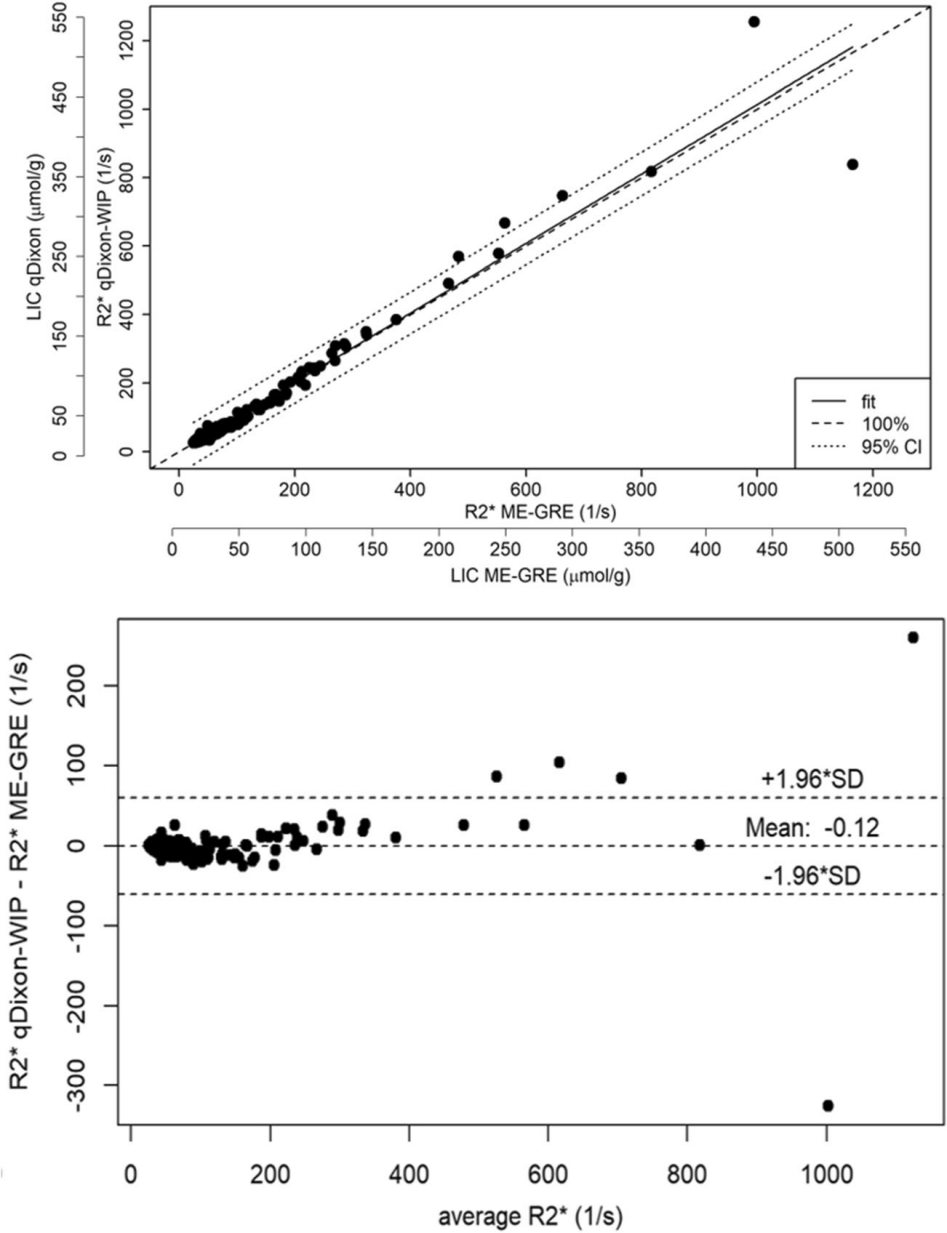
Correlation between liver R2* of R2-ME-GRE and qDixon-WIP (top) and corresponding Bland-Altman plot (bottom) (mean difference = − 0.125; SD = 30.667; concordance correlation coefficient: 0.976). qDixon-WIP was not limited to a maximum R2* value. The additional axis in the upper part of the figure enables the quantification of LIC based on the respective calibration equations given in the “Results” section

**Fig. 3:**
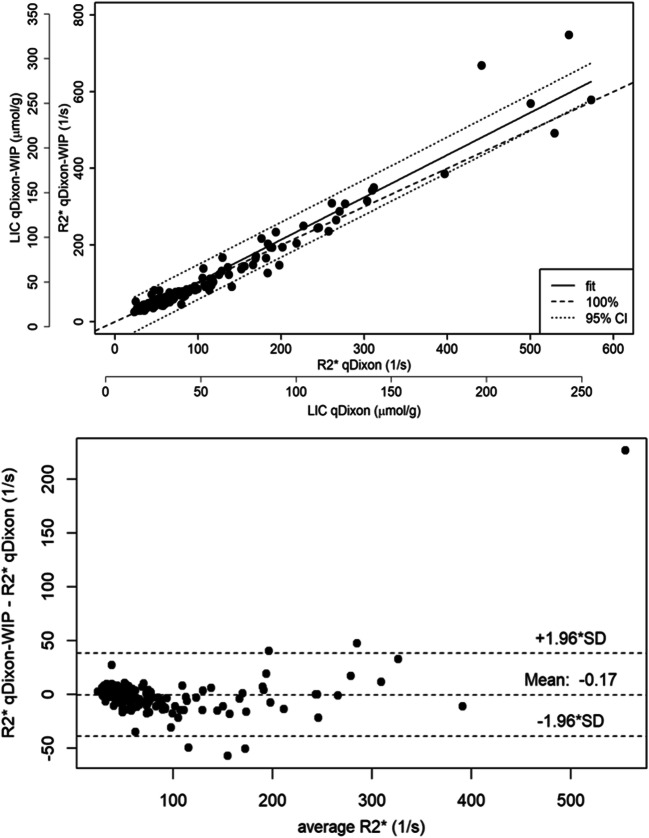
Correlation between liver R2* of qDixon and qDixon-WIP (top) and corresponding Bland-Altman plot (bottom) (mean difference = − 0.173; SD = 19.654; concordance correlation coefficient: 0.96). Only patients were compared, where no F/W swap occurred for qDixon. F/W swaps were completely absent for the improved variant qDixon-WIP. The additional axis in the upper part of the figure enables the quantification of LIC based on the respective calibration equations given in the “Results” section

The correlation between qDixon-WIP and R2*-ME-GRE was 0.95 (*p* < 0.05) and between qDixon and qDixon-WIP 0.95 (*p* < 0.05). Bland-Altman showed no relevant difference between qDixon-WIP and R2*-ME-GRE (mean = − 0.125; SD = 30.667) and between qDixon-WIP and qDixon (mean = − 0.173; SD = 19.654) (Figs. 2 and 3). In both cases, agreement was also substantial with concordance correlation coefficients of 0.976 (range: 0.969–0.981) and 0.96 (range: 0.949–0.969), respectively.

Results of the LIC-based analysis for qDixon are provided in Tables 2, 3, 4, and 5. Based on a simple yes/no decision (Table 2) as well as EASL classification (Table 3) concerning pathologic LIC, we found strong to almost perfect [26] agreement among all calibration formulas (see Table 4; overall agreement 83–100%, Cohen’s kappa: 0.83–1). Only for the EASL classification, the overall agreement between the calibration of Garbowski and Hankins was < 90%, while in all the other cases, an agreement of > 90% was found. In particular, regarding EASL classification maximum disagreement was always at most one severity class. For direct LIC quantification, the concordance correlation coefficient (Table 5) ranged from 0.942 (moderate agreement) to 0.999 (almost perfect agreement). The agreement of LIC values between our reference sequence and qDixon was almost perfect with a concordance correlation coefficient of 0.996.

**Table 2:** LIC analysis with overall agreement based on a simple yes/no decision concerning pathologic iron overload (LIC > 36 μmol/g)

	Overall agreement	Agreement in detail (no. of patients)		
		“Yes”	“No”	No agreement
Henninger^1^ versus Wood^2^	100%	134	252	0
Henninger versus Garbowski^3^	98.45%	134	246	6
Henninger versus Hankins^4^	92.75%	106	252	28
Garbowski versus Wood	98.45%	134	246	6
Garbowski versus Hankins	91.19%	106	246	34
Hankins versus Wood	92.75%	106	252	28

^1^Used formula: Fe (μmol/g) = 0.436 * R2* + 4.964 [9]

^2^Used formula: Fe (μmol/g) = 0.455 * R2* − 3.617 [6]

^3^Used formula: Fe (μmol/g) = 0.573 * R2* − 2.507 [20]

^4^Used formula: Fe (μmol/g) = 0.502 * R2* − 8.145 [19]

**Table 3:** LIC analysis with overall agreement based on the EASL classification [21]

	Overall agreement		Agreement in detail (no. of patients)				
				Group 0	Group 1	Group 2	Group 3
				Henninger			
Henninger^1^ versus Wood^2^	99.48%	Wood	Group 0	252	0	0	0
			Group 1	0	110	0	0
			Group 2	0	1	22	0
			Group 3	0	0	1	0
				Henninger			
Henninger versus Garbowski^3^	93.26%	Garbowski	Group 0	246	0	0	0
			Group 1	6	96	0	0
			Group 2	0	15	18	0
			Group 3	0	0	5	0
				Henninger			
Henninger versus Hankins^4^	92.23%	Hankins	Group 0	252	28	0	0
			Group 1	0	82	0	0
			Group 2	0	1	22	0
			Group 3	0	0	1	0
				Garbowski			
Garbowski versus Wood	93.78%	Wood	Group 0	246	6	0	0
			Group 1	0	96	14	0
			Group 2	0	0	19	4
			Group 3	0	0	0	1
				Garbowski			
Garbowski versus Hankins	86.53%	Hankins	Group 0	246	34	0	0
			Group 1	0	68	14	0
			Group 2	0	0	19	4
			Group 3	0	0	0	1
				Hankins			
Hankins versus Wood	92.75%	Wood	Group 0	252	0	0	0
			Group 1	28	82	0	0
			Group 2	0	0	23	0
			Group 3	0	0	0	1

^1^Used formula: Fe (μmol/g) = 0.436 * R2* + 4.964 [9]

^2^Used formula: Fe (μmol/g) = 0.455 * R2* − 3.617 [6]

^3^Used formula: Fe (μmol/g) = 0.573 * R2* − 2.507 [20]

^4^Used formula: Fe (μmol/g) = 0.502 * R2* − 8.145 [19]

**Table 4:** Cohen’s kappa values for agreement of Tables 2 and 3

	Cohen’s kappa for yes/no decision	Cohen’s kappa for EASL classification
Henninger versus Wood	1.0 (Std. Err: 0.0)	0.989 (Std. Err: 0.007)
Henninger versus Garbowski	0.966 (Std. Err: 0.014)	0.866 (Std. Err: 0.024)
Henninger versus Hankins	0.832 (Std. Err: 0.03)	0.832 (Std. Err: 0.029)
Garbowski versus Wood	0.966 (Std. Err: 0.014)	0.877 (Std. Err: 0.023)
Garbowski versus Hankins	0.799 (Std. Err: 0.032)	0.717 (Std. Err: 0.034)
Hankins versus Wood	0.832 (Std. Err: 0.03)	0.843 (Std. Err: 0.029)

Cohen’s kappa < 0 no agreement, 0–0.20 slight, 0.21–0.40 fair, 0.41–0.60 moderate, 0.61–0.80 substantial, and 0.81–1 almost perfect agreement

**Table 5:** Concordance correlation coefficients for direct LIC values calculated by different calibration equations (concordance correlation coefficient < 0.9: poor agreement; 0.9–0.95: moderate agreement; 0.95–0.99: substantial agreement; > 0.99: almost perfect agreement)

	Concordance correlation coefficient	Mean difference (μmol/g)	Standard deviation of mean difference (μmol/g)
Henninger^1^ versus qDixon^5^	0.996 (0.995–0.997)	2.5	0.14
Wood^2^ versus qDixon	0.996 (0.995–0.997)	2.3	1.5
Garbowski^3^ versus qDixon	0.962 (0.961–0.963)	1.1	9.6
Hankins^4^ versus qDixon	0.942 (0.934–0.949)	10.3	4.7
Henninger versus Wood	0.999 (0.999–0.999)	0.2	1.3
Henninger versus Garbowski	0.959 (0.956–0.961)	3.6	9.5
Henninger versus Hankins	0.963 (0.957–0.967)	7.8	4.6
Garbowski versus Wood	0.969 (0.967–0.972)	3.4	8.2
Garbowski versus Hankins	0.947 (0.939–0.953)	11.4	4.9
Hankins versus Wood	0.967 (0.963–0.971)	7.9	3.2

^1^Used formula: Fe (μmol/g) = 0.436 * R2* + 4.964 [9]

^2^Used formula: Fe (μmol/g) = 0.455 * R2* − 3.617 [6]

^3^Used formula: Fe (μmol/g) = 0.573 * R2* − 2.507 [20]

^4^Used formula: Fe (μmol/g) = 0.502 * R2* − 8.145 [19]

^5^Used formula: Fe (μmol/g) = 0.434 * R2* + 6.135 (see “Results”)

**Fig. 4:**
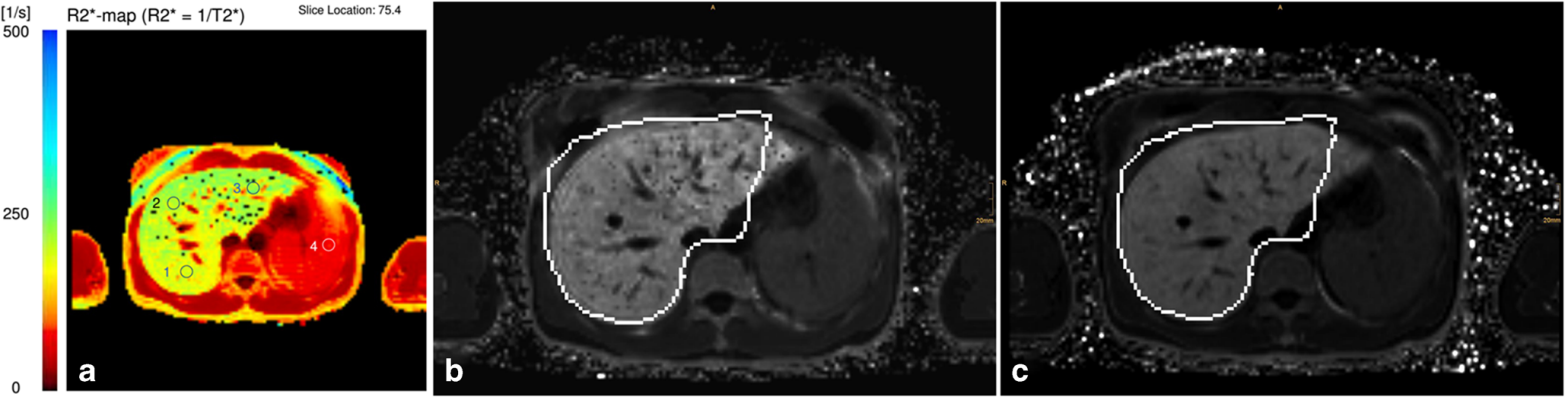
A 42-year-old male patient with known secondary hemochromatosis (thalassemia). R2*-ME-GRE (**a**) showed a R2* of 211.7 1/s, qDixon (**b**) 204.9 1/s, and qDixon WIP (**c**) 207.5 1/s. Results of all 3 sequences correlate very well with each other and show no clinically relevant deviations (the white outlines in **b** and **c** are liver outlines automatically detected for qDixon and qDixon-WIP during image reconstruction)

**Fig. 5:**
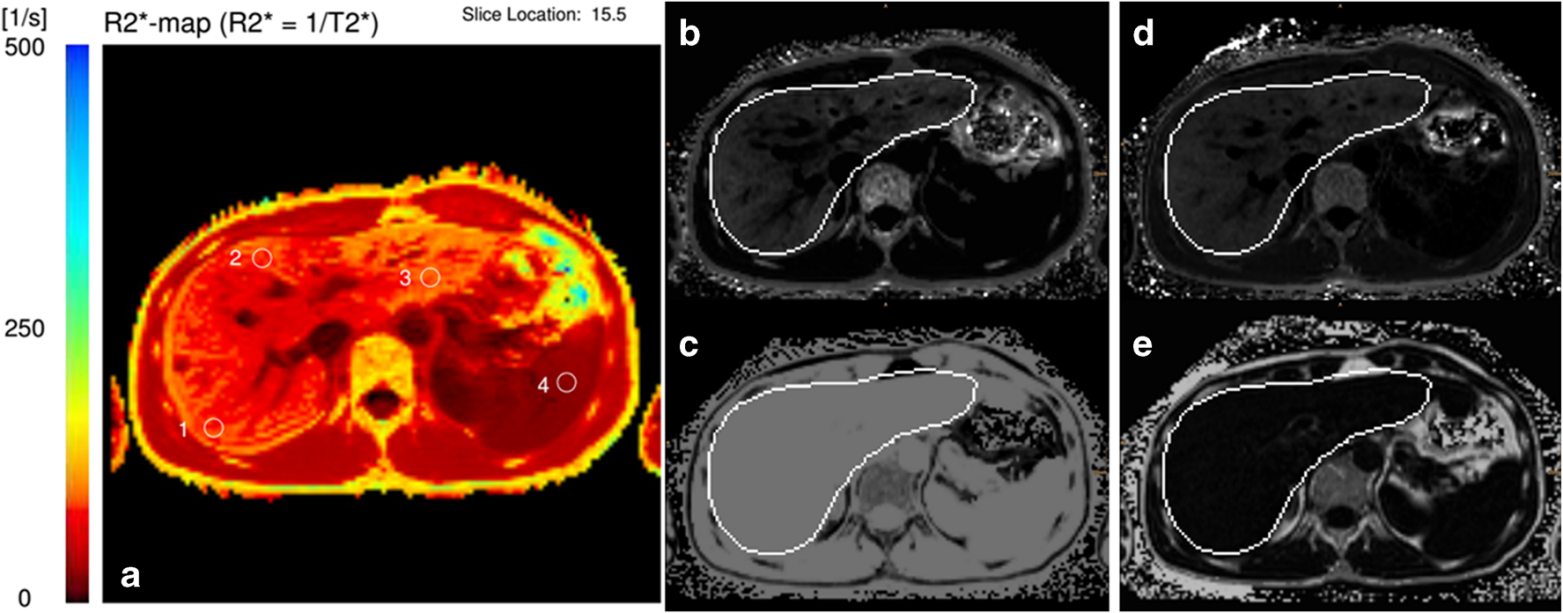
A 45-year-old male patient with suspicion of primary hemochromatosis. R2*-ME-GRE revealed a small increase of liver R2* with 83.6 1/s (**a**). qDixon showed a complete F/W swap with unusable results: the R2* was 37.2 (**b**) and the proton density fat fraction images (**c**) were not evaluable. qDixon-WIP (R2*-map in **d** and proton density fat fraction in **e**) was normally usable with a liver R2* of 79.3 1/s. Genetic analysis confirmed primary hemochromatosis (the white outlines in **b**-**e** are liver outlines automatically detected for qDixon and qDixon-WIP during image reconstruction)

General patient examples are provided in Figs. 4 and 5.

## Discussion

In this study, the qDixon sequence has been proven as a reliable approach for the calculation of hepatic iron in daily clinical routine. In general, our results showed an excellent agreement between qDixon and our reference sequence. This excellent agreement thereby cannot be automatically assumed, as the used methods differ in several technical aspects like 2D versus 3D acquisition mode, number of acquired echoes, significantly different echo times (especially initial TE), and the used post-processing algorithms (inline Dixon water/fat separation with multifat peak modeling vs. offline truncated exponential fit).

Further, we showed that the improved version qDixon-WIP delivered far more robust results than the original sequence: we encountered no F/W swap with qDixon-WIP, and our results were not limited to a maximum R2* value. The R2* values of qDixon-WIP also had an excellent agreement with values from our reference sequence (*r* = 0.95).

In contrast to the qDixon-WIP, the current version of qDixon does not deliver maps in LIC units; the operator is still required to use a formula from the literature to convert R2* to LIC [9, 20], which is frequently required by the referring clinician. In addition to cross-calibration with our reference sequence, we compared different calibration equations from literature to obtain LIC values based on the qDixon sequence. Thereby, we found the highest agreement between the calibrations by Wood et al and Henninger et al. Based on a simple pathologic iron yes/no decision, only the overall agreement between the calibration of Hankins and our cross-calibration for qDixon was < 90%. For all other calibration equations, agreement was always > 90%. The agreement for EASL severity classes was < 90% only between the calibration of Hankins et al and Garbowski et al and the calibration of Hankins and our cross-calibration. It was > 90% for all other cases. In case that no cross-calibration is available, our LIC-based results cannot give a direct recommendation for the ideal calibration equation, but show that agreement among the different equations is very high and the differences in the various LIC results are small. This was also shown in the fact that using the EASL classification, only changes of at most one severity grade were found. Therefore, any of the calibration curves applied in this work can reliably be used for LIC quantification with the qDixon sequence, but we should keep in mind that changing the equation in the follow-up process during therapy can lead to wrong decisions in clinical management.

The study by Serai et al evaluated a 3D multiecho Dixon-based imaging sequence (mDixon) in a pediatric and young adult population [27]. They compared a commercially available mDixon sequence with a conventional GRE-based relaxometry. In agreement with our study, they found no statistically significant difference in T2* values between the two sequences. The main differences to our study are the patient population and size and the different sequence parameters. Further, in contrast to our study, the used reference sequence was not calibrated by liver biopsy and no correlation analysis concerning the LIC and the use of different calibration curves was applied.

Jhaveri et al compared a R2* sequence, similar to our qDixon-WIP, with the R2 FerriScan method [12]. They observed that both provide equivalent quantification of the LIC within the limits of random uncertainty and concluded that iron heterogeneity is the primary source of the uncertainty. One limitation of this study was that ROIs could not be co-registered between the two techniques, which lead to uncertainties. In our study, we used a different reference sequence and manually co-registered ROIs between the different sequences. We observed an excellent agreement among all three sequences.

Surprisingly, we also found an excellent agreement between qDixon and qDixon-WIP, although the initial TE of both sequences differs markedly with a long TE of 2.38 ms for qDixon and a short TE of 1.04 ms for qDixon-WIP. This may be an indication for the appropriateness of the combined signal model containing both PDFF and R2*, which should minimize the impact of acquisition settings on the results. The longer TEs in qDixon are likely the cause for the observed upper R2* limit of approximately 400 1/s. Further, both the qDixon-WIP and our reference sequence R2*-GRE-GRE have an almost identical initial TE which could be the reason for the slightly better correlation between these two sequences.

One limitation of our study is the reference sequence employed. Its implementation, using fat saturation and a particular fitting procedure, is only one of many options, but this is also the case for most other R2* relaxometry methods that were correlated with histopathology. In this context, it has to be pointed out that the used reference method was calibrated by means of biopsy in an earlier study [9] and is now already used at our hospital successfully for years in daily clinical routine. Confidence in the method has reached such a level that our clinical partners usually do not perform liver biopsies anymore. In this respect, biopsy of the liver with histopathology is no longer considered justifiable due to the known drawbacks [1, 28–30]. Another limitation is that we only had the possibility to evaluate one vendor solution, which may raise the question of vendor bias. Since only MR scanners from a single vendor are used in our hospital, a multi-center study would be necessary to compare the different vendor solutions including “IDEAL-IQ” from General Electric, “StarQuant” (or mDixon-Quant) from Philips and “LiverLab” (or qDixon) from Siemens Healthcare. As this was far beyond the scope of this study, inter-scanner reproducibility was not investigated. Further, we did not focus on the evaluation of fat, which is also possible with qDixon and the original focus of this sequence.

## Conclusion

qDixon with 1.5 T is a reliable and exact method to quantify hepatic iron. Improvements of the implementation promise to eliminate its minor drawbacks of occasional F/W swaps, its limitation to R2* values of about 400 1/s, and missing inline LIC calculation.

## Abbreviations

EASL: European Association for the Study of the Liver
F/W swap: Fat-water swap artifact
LIC: Liver iron concentration
ME-GRE: 2D multigradient-echo sequence
MRI: Magnetic resonance imaging
PACS: Picture archiving and communication system
PDFF: Proton density fat fraction
qDixon: 3D-multiecho-Dixon sequence with inline calculation of PDFF and R2*
qDixon-WIP: Improved version of the 3D-multiecho-Dixon implementation
ROIs: Regions of interest
